# Optimizing weight loss outcomes for bariatric surgery patients: the role of physical activity

**DOI:** 10.1186/1753-6561-6-S4-O1

**Published:** 2012-07-09

**Authors:** R Boddu, E Wilson, B Snyder, TD Wilson

**Affiliations:** 1 University of Texas, Houston, USA

## Introduction

The effect of physical activity on the weight loss outcomes post bariatric surgery is not well understood. This study set out to determine if there was a correlation between physical activity levels of patients and their percent excess weight loss following bariatric surgery.

## Methods

Utilizing the bariatric clinic’s database 261 participants were identified who underwent bariatric surgery between June 2008 and June 2009. GPAQ (version 2) questionnaire developed by the WHO was chosen as the tool to assess and quantify the physical activity levels. In accordance with the GPAQ guidelines, the MET minutes and the total physical activity levels were calculated. Percent excess weight loss values found in the clinic database were gathered from the follow up visits of the participants.

## Results

A total of 125 (57.0 %) patients participated in the study. The demographics being, mainly female (258; 98.8%) and Caucasian (148; 56.7%). Surgeries included predominantly lap-band (110; 42.1%); roux-en-y gastric bypass (94; 36%); and sleeve (20; 7.6%). When stratified by low, medium and high physical activity levels, all three groups showed an increasing percent excess weight loss over time during the six months postoperative period (mean: 17.99 to 20.02 at one month versus mean 35.93 to 40.67 at six months). However, there was no correlation between the physical activity levels or the MET-minutes and the percent excess weight loss in the study group as a whole or when stratified by the surgery type.

## Conclusions

Our cross-sectional study failed to demonstrate a correlation between physical activity levels and percent excess weight loss in post bariatric surgery patients. Further prospective studies including other variables such as, percent fat loss, lean body mass values, preoperative physical activity levels, and caloric intake are recommended to gain a better understanding of the role played by physical activity levels in percent excess weight loss outcomes of bariatric patients.

